# Environmental cues for healthy food marketing: The importance of in-store research into three conversions

**DOI:** 10.3389/fnut.2022.1078672

**Published:** 2022-12-21

**Authors:** Nils Magne Larsen, Valdimar Sigurdsson, Didrik Gunnarsson

**Affiliations:** ^1^Department of Business and Economics, UiT The Arctic University of Norway, Harstad, Norway; ^2^Department of Business Administration, Reyjavik University, Reykjavik, Iceland

**Keywords:** impression management, environmental cues, in-store research, healthy choices, floor displays, nostalgia, music, in-store advertising

## Abstract

Since retailers control the space where consumers tend to make the vast majority of their food purchase decisions, they can take measures to promote healthy living. Increasing relative sales of healthy food can contribute to the ongoing battle against preventable lifestyle diseases. We show how retailers can use impression management and environmental cues in their stores to influence consumers' sales responses to healthy food. This paper advocates in-store research in this realm and introduces three consumer behavior levels - reaching, stopping/holding, and closing the sale - as micro-conversions when retailers use impression management on their consumers. We showcase impression management at each conversion level by testing the effects of placing healthy and unhealthy food items on a floor display in the store area with the most traffic, with or without background music and an advertisement. The results demonstrate that a healthy food product can outperform the sales of popular unhealthy foods. The floor display, for example, increased the sales of the targeted “healthy product” by 570% on average during the intervention periods, compared with the baseline. We discuss the importance of in-store research into three conversions to enable further development of impression management and the use of environmental cues for healthy food promotion.

## 1. Introduction

Healthy food consumption is important for chronic disease prevention and weight control (1). Humans have evolved so that most prefer energy-dense foods and sugar (2). Overconsumption of energy-dense food served well in the past, as people did not have access to food resources as readily as they do nowadays with modern grocery stores. This has led to mismatches between ancestral conditions and those found in the present world (3). Obesity has reached epidemic proportions in many Western societies, and the risk for lifestyle diseases like hypertension, diabetes, heart problems, and other significant health issues has increased substantially (4, 5). Worldwide, 40% of adults are overweight or obese, and unhealthy food choices are one of the culprits (6). It can be said that food retail practices are partly responsible for this undesirable situation (7). A concept related to the rise in obesity is “obesogenecity” of the environment. Obesogenicity is the “sum of influences that the surroundings, opportunities, or conditions of life have on promoting obesity in individuals or populations” [(8) p. 564]. The general advice in governmental policies, however, is to eat less sugary and fatty foods and more fruit and vegetables, lean meats, fish, and wholegrain cereal foods (9, 10). As such, there is a need to discern and articulate cues in the retail environment to guide healthier options.

As corporate and product brands have personalities (e.g., The Explorer - Red Bull), the same principles guide their relationship with consumers as the principles of interpersonal communication (11, 12). As such, retailers and food brands can use impression management strategies to create the desired image of themselves in the eyes of consumers [e.g., through celebrity endorsement (former England footballer Gary Lineker and Walkers Crisps)]. Much in-store marketing falls under impression management: techniques to establish meaning for interactions and to guide actions, often through dramaturgical ways (13–17). Impression management stems from social psychology and has been applied in various contexts within marketing and communications [see, e.g., Angulo-Ruiz et al., Harris and Spiro, Schlenker, Schniederjans et al. (18–20)].

Consumers in general, at least in the Western world, are faced with a far greater selection of food than ever before, and many can afford to try out new dishes. At the same time, there have been changes in the perceptions of eating. Eating has become more of an experience in which food is consumed for pleasure rather than being simply a way to provide the body with nutrients and replenish energy (21). Many brands now define themselves by a reference to health, environment, society, and culture as more consumers buy and use food to signal who they are, what they represent, and who they want to be (22). The importance of experiences and pleasure also suggests that healthy food should, to a greater extent, be marketed in stores using multi-sensory displays to help consumers imagine what it will feel like to eat it (23).

Food is constantly becoming more fragmented into health-damaging junk food vs. health-promoting food. Although unhealthy food is becoming the “new tobacco,” with increased media and political monitoring, the tactics to promote such food have been successful. These include using the store as a medium, with good in-store placements, images, symbols, and characters to impress and evoke the desired response. For instance, American Idol judges are always seen behind Coca-Cola cups as product placement/branded entertainment. This design and these placements have been identified under various conditions in recent decades and are supposed to influence consumers into buying these products, which tend to be unhealthy. The marketers (or, in our context, the retailers) are applying impression management, using signals and cues to make an impression on the customers, and portraying the product in a certain light—literally or figuratively (13). The brands try to enhance sales through in-store environmental cues, such as floor displays in trafficked areas, which is a common and effective practice for potato chips, as an example (24). Other techniques include in-store advertising for chocolate (e.g., “Melts in your mouth, not in your hands” - M&M chocolate), sounds of opening and pouring a coke (25, 26), or using nostalgic music (e.g., “I'd like to teach the world to sing…” - Coke song, 1971). The overall purpose of our research program, for the past 15 years, has been to test how in-store marketing tactics, such as environmental manipulations and impression management, can turn this unhealthy food marketing around to help consumers choose healthy, sustainable fish products with environmental/retail signaling [see e.g., Sigurdsson et al. (27–29)].

The primary aim of the current paper is, therefore, to introduce and assess how and to what extent it is possible to use in-store research (30–33) to study the effects of value-adding impression management techniques on consumers' brand choices in their actual environments. We showcase impression management and environmental cues on the main customer route, the so-called “racetrack” or perimeter—the wide pathway circling the store floor, for healthy food promotion. This is done with the aim of using the most effective area of the store to influence the shoppers' impressions in terms of noticing, stopping, and buying healthy food. By adopting impression management responsibly, retailers can use environmental cues to nudge consumers to make healthier and more nutritious choices. They can impress consumers directly (e.g., by increasing the focus and image of healthy food through placements, sounds, or by appealing to nostalgia, local values, or history) or indirectly by better understanding consumers' own impression strategies (how consumers use products as props when impressing others). Goffman's (14) theory on impression management is that individuals put on a performance to influence other peoples' views of them in social situations and that they can use food as items or props in that show (3). In the same vein, retailers organize their stores to influence and frame purchasing and consumer habits (34). For example, they locate fruit and vegetables at the entrance to create an association of freshness, and the bakery further inside to evoke appetite. Furthermore, when judging the caloric content of food, consumers tend to base their judgment on seemingly superficial environmental cues (35).

Goffman (14) discusses the “setting,” which is comprised of the items and props that set the scenery for the interaction, and Schlenker (17) defines impression management as an attempt to shape impressions of a person, a group, an object, an event, or an idea. This fits well with retailers' in-store marketing strategies, which aim to regulate consumers' minds by affecting how consumers move around, use their eyes, and perceive the nature of choice (34). Retailers, therefore, attempt to shape the consumers' impressions influenced by a product, and this can happen with or without conscious awareness of it (36). In this paper, we will:

- Show how impression management and environmental cues are used in stores by brands and retailers to influence consumers' responses toward unhealthy food. We will give several examples and discuss how this marketing can be “turned around” to be more focused on active retailing of healthy food.
- Introduce the three levels of in-store marketing analysis as conversions for impression management. That is: (1) exploit consumers' dominant path (reach), (2) placements that impress consumers (stop/hold impressions), and (3) product attribute signaling (closing the sale).
- Give research examples at each level of in-store marketing analysis and showcase impression management involving floor displays, background music—as sensory marketing—and nostalgic advertising along the perimeter of a store (a wider pathway circling the store floor).

We agree with Sorensen (37) that retailers should go from passive to active roles— guiding and impressing consumers. However, we like to add that this active role increases the retailer's responsibility. It, therefore, needs to focus on healthy and sustainable environmental cues and impressions: responsible active retailing.

## 2. In-store impression management for healthy food marketing: Three conversions

Research suggests supermarkets tend to over-promote low-nutrition food. For instance, Cohen et al. (7) found that food stores used end-aisle displays, special floor displays, and checkout displays to promote sugar-sweetened beverages, candy, salty snacks, and sweetened baked goods. When promoted at the point of purchase, the sales performance of unhealthy vs. healthy products can affect the retailer's allocation of promotional floor space to healthy vs. unhealthy products in the areas of the store with the most traffic. The double conversion concept by Sorensen (37) suggests that retailers should monitor how effective different products are in converting a visitor of a zone into a shopper (a visitor that becomes engaged) and converting the shopper into a purchaser. That is, to measure a product's stopping/holding power (converting into a shopper) and closing power (converting into a purchaser). His guidelines for placing products further suggest that product leaders (products or categories with great closing and stopping/holding powers) should be put in very high-traffic locations (reach) and given priority in secondary placements, such as in the perimeter (37). In order to determine the effect of reach, and the stop/hold and closing power of various healthy products compared to unhealthy products, experimental efforts are a necessity. Experiments are needed where similar displays of various healthy and unhealthy products are tested and examined in a high-traffic location of the store (reach). Observational data (38) should then be examined to assess stop/hold and closing power in more detail. Transactional data should then be gathered to assess the performance of the various displays in terms of total sales and sales increases (compared to baseline; that is, the total sales when the products have their usual placement in the store).

### 2.1. Retail cues for reaching and stop/holding consumers, closing the sale

Since retailers tend to make energy-dense, nutrient-poor foods and beverages more readily available to shoppers than healthier food, grocery stores tend to be obesogenic (39). This means that retailers commonly place sweets and other products with high glycemic carbohydrates in highly visible areas. Because of exposure's importance in retail, healthy food could be more clearly exposed in the retail environment to promote them more actively. This is a prerequisite for in-store impression management for healthier food, as out of sight usually means out of mind.

Grocery stores play a significant role in food purchasing (30). They offer a promising venue for various efforts to improve consumers' health through better nutrition (40). If retailers do not voluntarily act more responsibly, they risk facing more substantial governmental restrictions on their operations. A recent example is from the UK where “The Food (Promotion and Placement) Regulations 2021” (41), effective from October 1 2022, will restrict the placement of less healthy food and drinks in stores having 185.8 m2 (2,000 sq ft) or greater of “relevant floor area” (42). This new regulation limits what retailers can do to make less healthy food and drinks “too easily” available to shoppers, as it regulates which items cannot be placed in the main customer route through the store, including the entrance and the checkout area. This paves the way for better placement of healthier food inside stores and demands more research on impression management strategies and environmental cues as focal factors in healthy food promotion.

Published research examining in-store migration patterns using path data has revealed that consumers rarely shop the entire store (31, 38, 43–45). Most shoppers walk around the store's perimeter and only visit the specific aisles they need (43), preferring wide, open spaces that allow them to circumnavigate the store (37). While consumers walk around the perimeter, they use physical products as external memory cues to trigger forgotten needs (46). As such, activating thoughts about healthy products is contingent upon shoppers noticing the healthy products in the retail setting while shopping (47). An effective strategy to promote the consumption of more healthy products would be to promote such products in areas where most consumers shop in the store, as the “best products” are put in the best placements (with the most exposure). That sends clear signals to consumers (48). For any brand, unhealthy or healthy, the retail store is the most crucial point to affect and impress consumers, as most brand decisions are made in the store (49). What consumers see is what they buy, and if they do not see a particular brand, it is the same as if it is not there (37). Thus, the store is a critical media; its many shelves and displays are media points with different costs and rating points (the percentage of the shoppers the point reaches during a given day or daypart). Sorensen (37) concludes that end aisle displays (end-caps) and freestanding floor displays get “the lion's share” of the in-store media exposure, based on the share of shoppers being reached by in-store media points, and the number of seconds for which the average shopper sees the media during their entire shopping trip. Impression management strategies should rely on empirical research into key metrics of continuous streams of in-store behavior. Those include store area coverage, shopping duration, travel distance, walking speed, shopper efficiency, and carrying equipment use. This is the foundation for an empirically grounded shopper behavior theory (31, 50), providing metrics and benchmarks that are useful for retailers to boost their performance (30, 31, 38).

Research demonstrates that healthy food promoted successfully by achieving the “right impression” at premium locations in the store can enjoy significant sales increases. In a study by Sigurdsson et al. (32), alternating treatment designs embedded in a multiple baseline design in two stores examined the effects of product placement and in-store advertisements on approximately 100,000 customers. That was done by replacing unhealthy items with healthy items at the checkout lines in an effort to grab attention and symbolize new healthier times at this “grand finale of the retailer's theatrical performance.” Such a simple environmental modification resulted in a decrease in the sales of the unhealthy items and an increase in the sales of the healthy items. Similarly, Payne and Niculescu (51) showed a significant increase in micro-pack purchases of fruits and vegetables placed at checkout displays. Their data further indicated that shoppers purchased the micro-packs instead of other food items. Ejlerskov et al. (52) also demonstrated such an effect. They found that the introduction of food policies in six UK supermarket chains that reduced the availability of less-healthy food at the checkout was associated with a 17.3% reduction in purchases of single-serve or small packages of sugary confectionery, chocolate, and crisps. Apart from increasing sales of healthier food items, healthy products at the checkout can also function as an environmental cue to control or modify the retailers' image or personality (e.g., that the store cares about its customers). The literature suggests that store environmental cues offer reliable information about a retailer and that displays can drive image valuations. Cornelius et al. (53) found that different storefront displays carry different image potentials. Here, the part of the impression management framework where organizational behavior aims to control or modify audience image (54) was applied in retailing. Environmental cues can also be employed to enhance the image and evaluations of individual brands. Buchanan et al. (48) demonstrated that consumers have a schema regarding displays, and displays that exist separately from traditional aisle shelves in particular (e.g., that important brands are given precedence in the store). They argue that consumers interpret separate displays as an indication of the brand's differentiation from other brands. In fact, where merchandise is located in the store can have a pronounced impact on consumer perceptions and behavior. Furthermore, Desai and Ratneshwar (55) showed that consumers perceive low-fat variants of junk food differently if they are located in a health-food section compared to a location among less healthy junk food of the same category.

## 3. In-store impression management for healthy food: Sensory marketing and nostalgia

Sensory marketing is the field of using various senses (hearing, tasting, vision, smelling, and touching) to send signals to consumers to influence their behavior (56). Overall, studies of background music have reported very different results as there are several variables at play, including the tempo, genre, volume, and more (57). As summarized by Biswas et al. (58), research has shown that music played at a comfortable level has a positive and relaxing effect on people. Excitement or stress tends to lead people to choose and consume unhealthy food and, contrastingly, low-volume ambient music enhances the likelihood of choosing healthy food options (58). To our knowledge, the experimental use of background music to create thoughts and associations has not been examined in the literature as a technique for sales increases despite all emphasis on the experience economy or marketing to the senses (59). The impact of music on consumer behavior in a retail setting has mainly been studied in terms of its effect on the shoppers' mood, length of shopping time, appraisal of store offerings, etc. Furthermore, of the relevant field studies completed in supermarkets, the vast majority used background music for the entire supermarket [e.g., Herrington (60), Vida et al. (61)], not as background music only played in close vicinity to, for instance, an in-store display for a healthy food item. As music itself has the ability to create thoughts and associations, and to change emotions (62), it can enhance customer perception of a brand. Therefore, there is a need to study the effects of background music in relation to in-store impression management.

Research shows that appealing to nostalgia can influence consumer motivations and behaviors. Nostalgia has been defined as a preference (liking, positive attitude, or favorable effect) that consumers have for things that used to be more common (e.g., popular or widely circulated) when they were young (63). Nostalgia arising from a consumer's memory can be activated by marketing stimuli and can evoke pleasurable cognitive responses that lead to a behavioral reaction, such as an impulse purchase (64, 65). In this vein, a brand or a specific product in the product line of a particular brand can serve as the stimulus for nostalgic reminiscences (64). The fact that many consumers wish to reexperience past times allows firms to use nostalgia in communication and product design to target and appeal cognitively and emotionally to potential customers (66). There are only a few studies dealing with nostalgia-related specificity of traditional food (67). Food is among the sensory stimuli most likely to take consumers back to their past by triggering memories, through thoughts such as “It is a long time since I ate this” or “I remember my mother serving me this for breakfast” (67–70). Nostalgia is, therefore, highly relevant in settings where traditional food is part of in-store impression management.

## 4. Showcasing in-store research into impression management

To be market-driven means seeing past the short-sighted and superficial inputs of customers, to gain a deep-down understanding that gives managers confidence their judgments are right … firms must be willing to continually learn and refine their judgments through broad scanning and experimentation. So if a company truly understands its present and prospective customers, it knows when to ignore the superficial reactions to a survey... [(71) p. 12].

When properly executed, field research provides a more accurate depiction of consumers' behavior than do studies set in laboratories or other artificial environments (72), and in-store behavior is in-store behavior, meaning that it needs to be studied in that situation and not only in a laboratory. Below, we showcase an example of an in-store experiment using impression management by testing the effects of placing an 800 gram can of fish balls produced by the brand manufacturer Vesteraalens in a floor display in the store area with the most traffic, with or without an advertisement (case study 1) and with or without background music (case study 2). The target brand, Vesteraalens, has a long and adventurous history, and the target product has been a traditional cuisine in Norway for more than a century. The brand uses history and appeals heavily to nostalgia as part of its market communication, evident from the content on its website (www.vesteraalens.no) and the use of black and white historical images on the top and bottom of the fish ball cans. The fish balls are preserved in an iconic green can and have a high content of fresh haddock (57%) and a low content of both calories (50 kcal per 100 g) and fat (0.2 per 100 g). It is an affordable and convenient meal, and healthier than many alternatives. Vesteraalens' category market share was as high as 70% in 2015 (73). Although being the leading brand among canned fish balls, it has lost some ground in the last three decades as many new alternatives for main meals have been introduced to consumers (e.g., tacos, tandoori chicken, lasagna, pizza). Hence, this product may be viewed as a niche product in the grocery store. At the same time, it might evoke nostalgic sentiments within older consumers and trigger positive memories from the past.

### 4.1. Case study 1

#### 4.1.1. Participants, settings, and materials

This case study was conducted at a discount store belonging to Coop in Norway. The target product chosen for this in-store experiment was Vesteraalens' 800 gram can of fish balls.

#### 4.1.2. Experimental design and procedures

The experiment was implemented using a group alternating treatment design, or ATD (74), comparing the behavior of consumers between different in-store interventions. The interventions included: a) the healthier target product displayed in the perimeter based on tonnage merchandising principles (a display technique in which large quantities of a product are displayed together, making it a focal point), and b) an in-store advertisement next to the display. Baseline comparisons were used in addition to the ATD as they provided further comparison and assessment of long-term effectiveness [e.g., Barlow and Hayes (75)]. This allowed for a later comparison of the baseline and the alternating experimental interventions. Consumers were not informed about the in-store experiment. Every day, the store was visited at least once by the study authors to keep records of the correct implementation of the experimental design.

#### 4.1.3. Treatments

The sequence of interventions was randomized and counterbalanced. A section of an ABCA pattern was used for the alternating treatments, where A represented the baseline/follow-up (the target product in its usual setting, the store shelves), B represented the target product displayed additionally in the perimeter of the store, and C involved displaying the target product additionally in the perimeter combined with an in-store advertisement. The display was a free-standing floor display of size 156 cm (width) x 117 cm (length). The in-store advertisement stated, “Vesteraalens fish balls have been on the dining table for more than 100 years.” The intent of this advertisement was not only to grab attention but also to act as an impression tactic or a motivational operation for consumer behaviors [see, e.g., Fagerstrøm (76)], with reference to local tradition and nostalgia.

#### 4.1.4. Response definitions and measurements

The dependent variable was sales of the target product as a proportion of the stores' total sales of grocery products (e.g., sales of the target product/gross store sales [including the target product]). Actual sales data were measured by the target product being scanned at the cash register when consumers bought it. The store was closed on Sundays, so the sales data were grouped into periods of 6 days of sales to compute the target product's sales as a proportion of the store's total sales. Each data point, representing either the baseline or one of the two interventions, had an equal number of days (6 days) and equal representation across days. The experiment was run for 78 days (13 x 6 days: 13 weeks), starting with baseline for 4 weeks, then 1 week of treatment B, 1 week of treatment C, 1 week with baseline, 1 week of treatment B, 1 week of treatment C, and finally 4 weeks with baseline as a follow-up.

#### 4.1.5. Results

As a characteristic of behavior analytical research (33), there is no emphasis on inferential statistics in the results section [see e.g., Baron (77), Hopkins et al. (78)]. Instead, we use visual inspections of graphs to interpret if there is a meaningful difference between conditions [see a critical discussion of this approach in (79)]. This is done as our experimental purpose is focused on diminishing behavioral variability between different interventions (80), with experimental techniques and replications instead of focusing on statistical significance.

**Figure 1:**
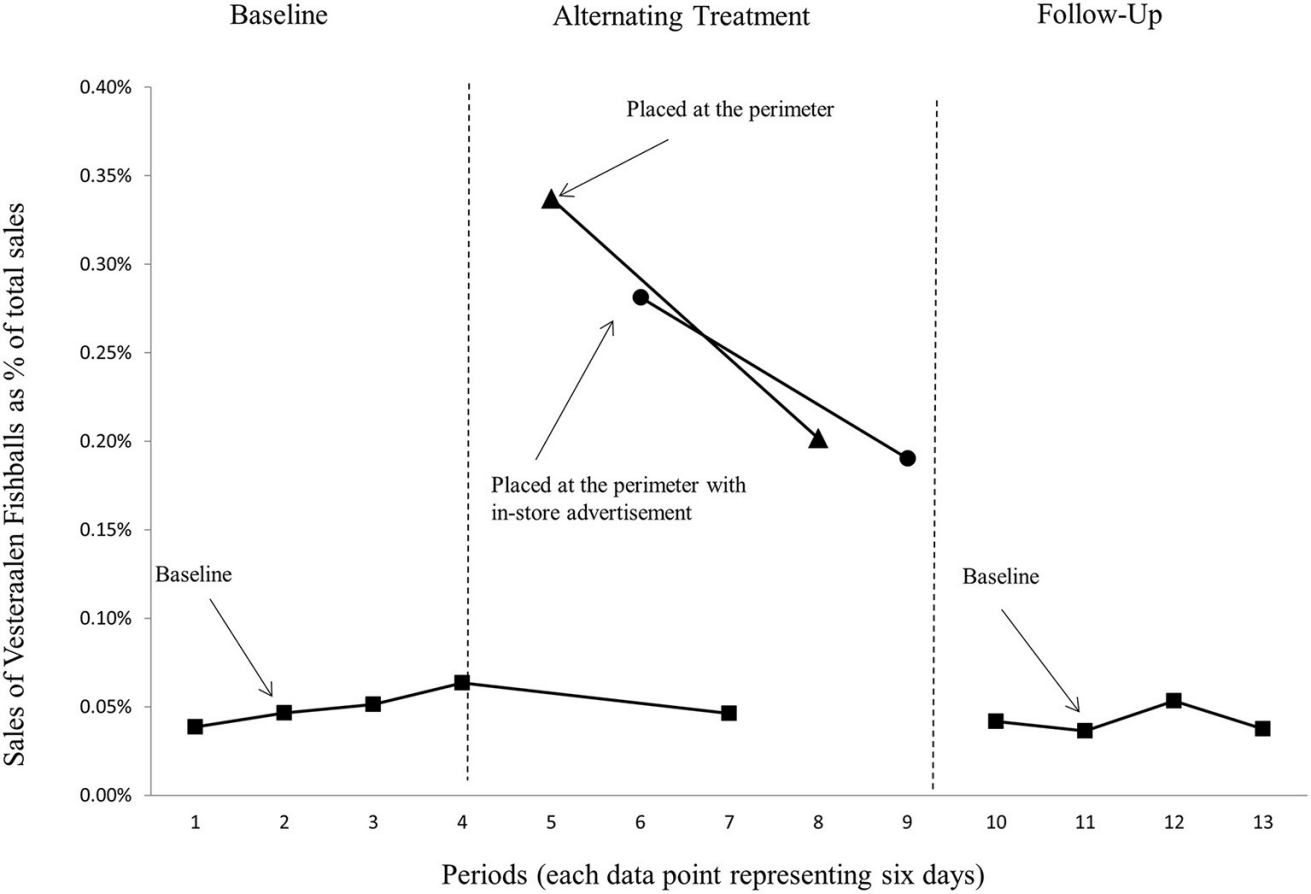
Sales of fish balls as a proportion of store total sales.

Figure 1 shows sales of the fish balls in percentage of total store sales, where each data point represents 6 days of sales statistics (average scores). Figure 1 shows that the baseline (the two graphs with squares representing the data points) is relatively stable (before, during, and after the treatments). This is when the target product was placed at its usual shelf location. A visual inspection of the graphs shows that displaying the fish balls in the perimeter of the store (in addition to its usual shelf location) was very effective. The graph representing baseline sales is considerably lower than the two graphs representing either a placement of the fish balls in the perimeter with no advertisement (the graph with triangles representing the data points) or a placement of the fish balls in the perimeter of the store with an ad (the graph with circles representing the data points). The relatively stable baseline demonstrates that considerable sales effects, stemming from placing the target product in the perimeter, had a low impact on baseline sales of fish balls. This indicates that the display, due to its location in the most trafficked area of the store (30, 31), made more consumers consider the target product (46, 47).

Based on a visual inspection of these graphs, we also conclude that the target product sales were not substantially different when the fish balls were placed at the perimeter with an advertisement compared to no advertisement. The two graphs intersect and have roughly the same downward slope. This suggests that the effect of placing the target product in the perimeter was diminishing and that the ad itself had little influence on this diminishing effect.

### 4.2. Case study 2

The experiments in case study 2 were implemented in a hypermarket store belonging to the same retailer as in case study 1. Here we examine whether the effects of a free-standing display of the healthy target product in case study 1 also transferred over to other types of retail formats. Since the advertisement in case study 1 showed no particular effect on purchase behavior, it was replaced with a treatment involving background music. The background music aimed to evoke associations with traditional food and thoughts of previous experiences with the healthy target brand. Case study 2 also involved two unhealthy target products to further assess the effectiveness of placing a healthier product in the most prominent area of the store, which usually promotes more unhealthy items such as chocolates, chips, and sodas. Finally, to enable better inference from the treatment involving increasing the reach of the target products by an additional placement in the store's perimeter, case study 2 also involved observing customer behavior.

#### 4.2.1. Participants, settings, and materials

The target products for the in-store experiments were the same 800 gram can of fish balls as in case study 1 (the “healthy/healthier product”) and a mix of large chocolate confections in bar form (chocolate bars - “the unhealthier products”) from the brand manufacturers Nidar (Unhealthy product A) and Freia (Unhealthy product B). The weight of the chocolate bars varied from 155 to 210 g. We chose Nidar and Freia large chocolate bars because of their high calories and fat content. For instance, a 200 g milk chocolate bar from Freia contains 550 kcal per 100 and 34 g fat per 100 g. Freia and Nidar are traditional Norwegian brands enjoying high market shares in Norway. The brand Freia is owned by Mondelēz International, while Nidar is owned by Orkla Confectionery & Snacks Norge.

#### 4.2.2. Experimental design and procedures

The experiments were implemented using a group alternating treatment design, or ATD (74), comparing the behavior of consumers between different in-store interventions. The interventions included: (a) the target products displayed in the perimeter based on tonnage merchandising principles similar to case study 1, and (b) music played in close vicinity to the healthy target product. Similarly to in case study 1, baseline comparisons were used in addition to the ATD. Consumers were not informed about the in-store experiments, and the store was visited at least once a day by the researchers to keep records of the correct implementation of the experimental design.

#### 4.2.3. Treatments

The sequence of interventions was randomized and counterbalanced. A section of an ABCA pattern was used for the alternating treatments involving the healthy target product, where A represented the baseline (the target product in its usual setting, the store shelves), B represented the target product displayed additionally in the perimeter of the store, and C involved displaying the target product additionally in the perimeter combined with background music. For both of the unhealthy target products, a section of an ABA pattern was used for the alternating treatment, where A represented the baseline and B represented the unhealthy target product displayed additionally in the perimeter. Although the free-standing floor display was of a smaller size than in case study 1 (due to differences between the stores, such as availability of floor space), the display size was similar for all the target products - 78 cm (width) x 117 cm (length). As background music, five songs by the Norwegian contemporary folk music artist Moddi were selected based on music-brand image fit [see e.g., Beverland et al. (62)]. The five songs were played close to the display of the healthy target product throughout the entire opening hours of the store (from 08:00 to 23:00 h). The music was played at a comfortable decibel level (55–60 dB.) measured by ATMO experts specializing in in-store music.

#### 4.2.4. Response definitions and measurements

The dependent variable was sales of each of the target products as a proportion of the stores' total sales of grocery products (e.g., sales of the healthy product/gross store sales [including the healthy product]), as well as average total sales per day in NOK [Norwegian currency kroner] of each of the target products throughout the experimental period. Actual sales data were measured by the target product being scanned at the cash register when consumers bought it. Similar to the store in case study 1, the hypermarket store was also closed on Sundays, so the sales data were grouped into periods of 6 days of sales to compute the target products' sales as a proportion of the store's total sales. Each data point, representing either the baseline or an intervention, had an equal number of days (6 days) and equal representation across days. Each experiment was run for 72 days (12 x 6 days: 12 weeks). The experiment with the healthy product (fish balls) had first 2 weeks with baseline, then 1 week of treatment B, 1 week of treatment C, 2 weeks with baseline, 1 week of treatment C, 1 week of treatment B, and then 4 weeks with baseline as a follow-up. The experiment with the unhealthy product A had first 1 week of treatment B, then 2 weeks with baseline, 2 weeks of treatment B, 2 weeks with baseline, 1 week of treatment B, and then 4 weeks with baseline as a follow-up. The experiment with the unhealthy product B had first 3 weeks of treatment B, then 2 weeks with baseline, 5 weeks of treatment B, and then 2 weeks with baseline as a follow-up.

To enrich the dataset, two trained research assistants conducted structured observations of customers passing the freestanding floor display of the healthier target product and the unhealthy target product B. The number of observations ranged from 300 to 457. We used a predesigned observational form with clearly defined behaviors (consumers entering the zone [reach], noticing, stopping/evaluating [stop/hold power], and buying [buying power] the target product), and occurred/not occurred as response alternatives.

#### 4.2.5. Results

**Figure 2:**
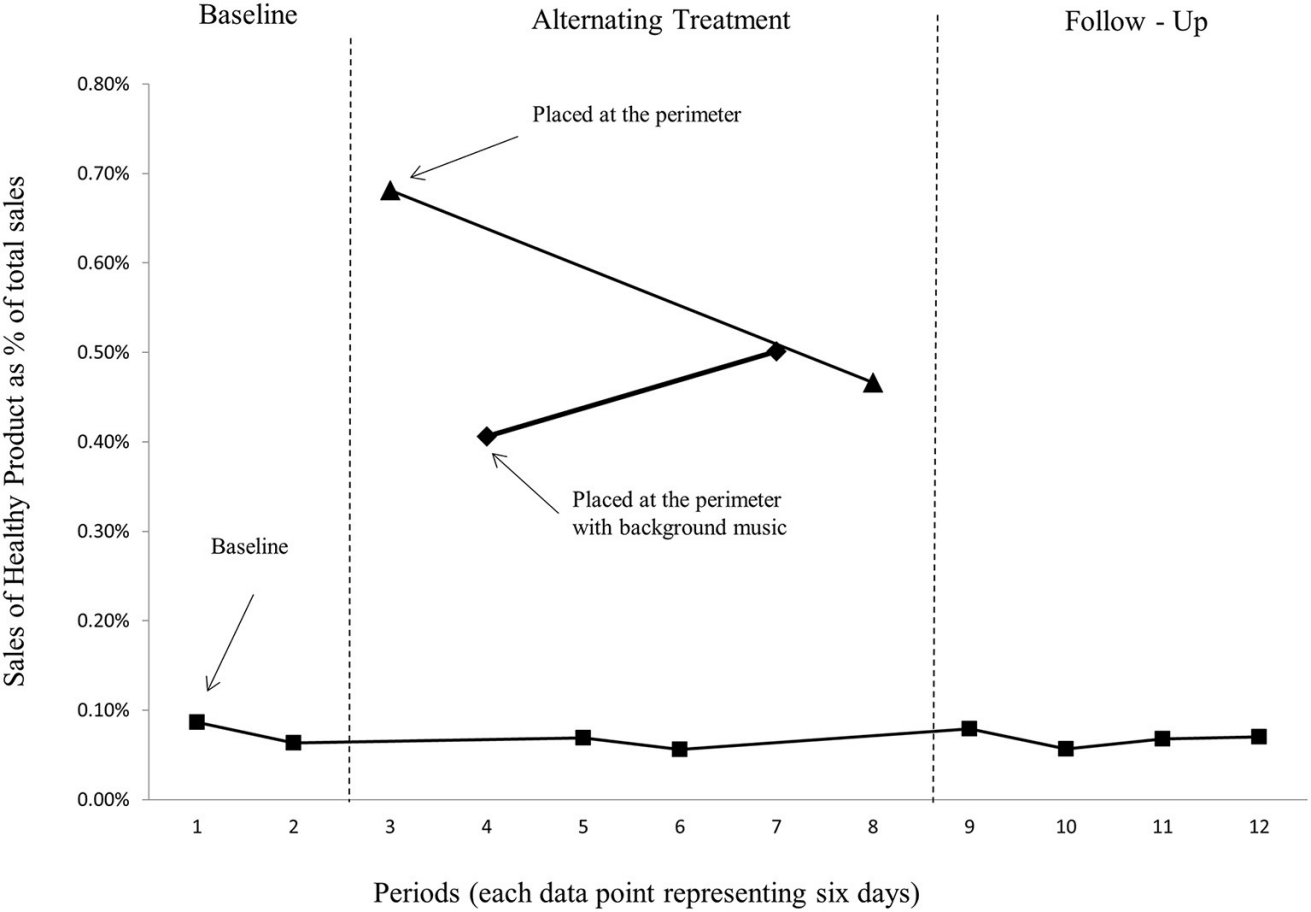
Sales of the healthy product (fish balls) as a proportion of store total sales.

Similar to in case study 1, the results section places less of an emphasis on inferential statistics and instead uses visual inspections of graphs to interpret if there is a meaningful difference between conditions. Figure 2 shows sales of the healthy target product (fish balls) in percentage of total store sales, where each data point represents 6 days of sales statistics (average scores). The results are very similar to those reported in case study 1. Again, the baseline (the graph with squares representing the data points) is relatively stable before, during, and after the treatments.

Figure 2 further shows that displaying the healthy product additionally in the perimeter of the store was very effective as the graph representing baseline sales is considerably lower than the graph representing the placement of the healthy product at the perimeter of the store (the graph with triangles representing the data points), but also that this effect diminishes over time. From the graphs, we can also detect that the presence of background music does not show substantially higher sales than merely placing the product in the perimeter without any music. As in case study 1, the relatively stable baseline demonstrates that considerable sales effects stemming from placing the healthy product in the perimeter had a low impact on baseline sales, which again indicates that the display, due to its location, made more consumers consider the healthy product (46, 47).

**Figure 3:**
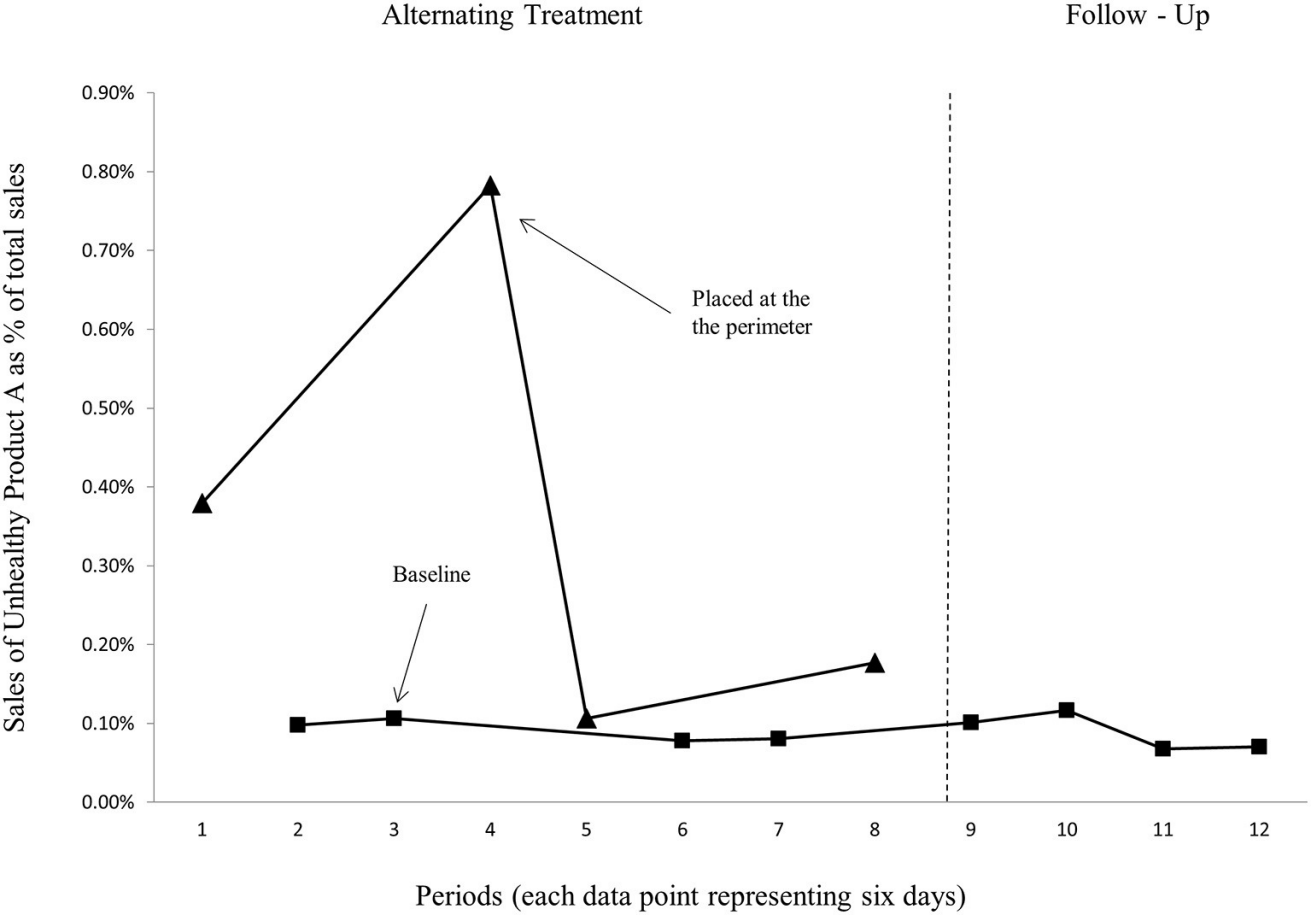
Sales of the unhealthy product A as a proportion of store total sales.

**Figure 4:**
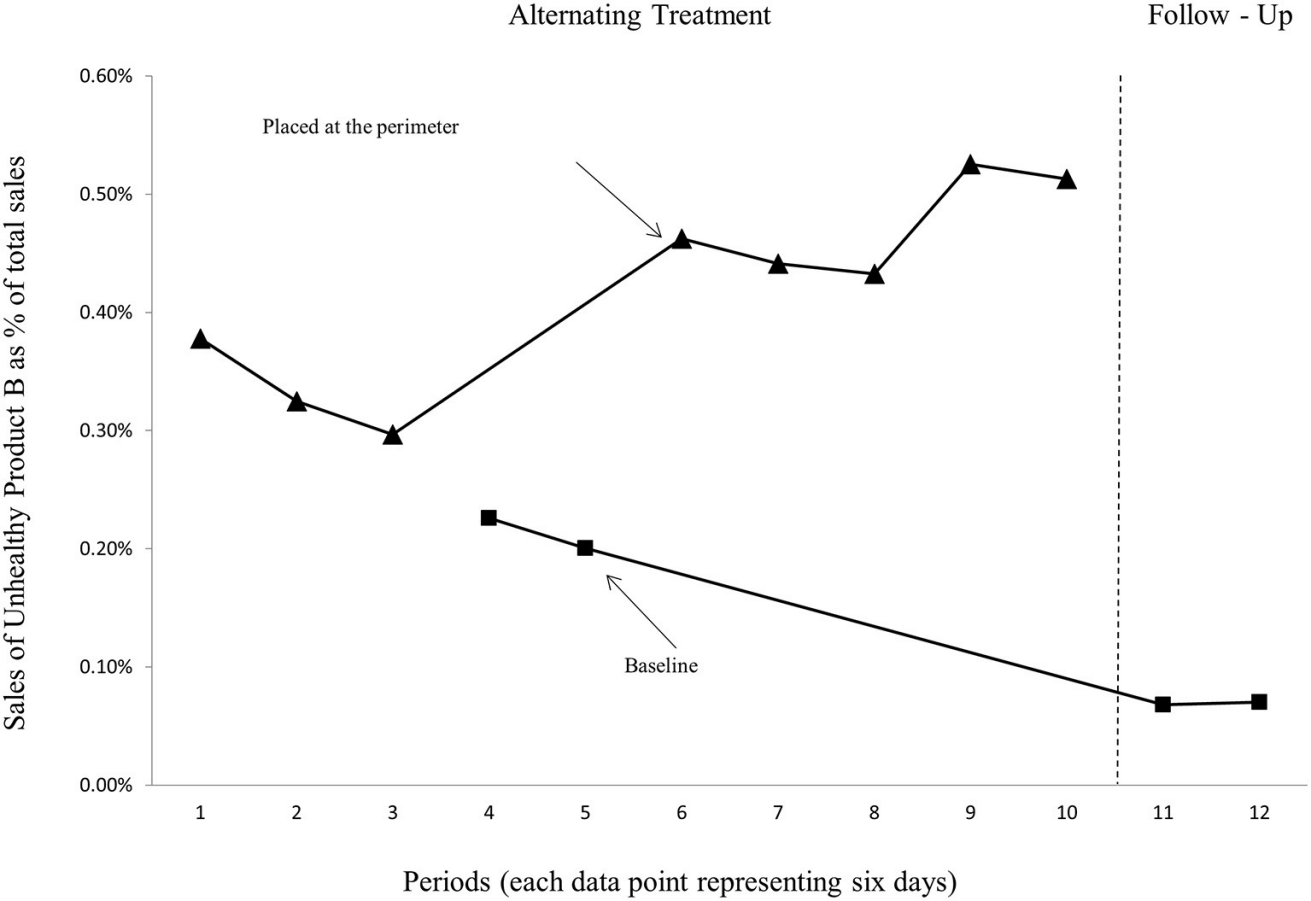
Sales of the unhealthy product B as a proportion of store total sales.

To further examine the effectiveness of placing healthier products in the most prominent area of the store that usually promotes more unhealthy items (7), we conducted additional experiments involving a mix of large chocolate confections in bar form from two leading brands, Nidar (Unhealthy product A) and Freia (Unhealthy product B). Figures 3, 4 show the sales of unhealthy products A and B, respectively, when the products were only placed at their usual shelf location in the store (baseline), and when they were also placed at the perimeter in a freestanding floor display.

Here, baseline data (taken prior to the interventions) is missing, and that reflects the challenges involved in studying actual behaviors with actual consumers in their real retail situations—it can be hard to get the data or follow a strict experimental design [see a discussion in Sigurdsson et al. (33)]. Based on a visual inspection of the graphs in Figures 3, 4, we conclude that the two unhealthy products have higher variability in average sales when placed in the perimeter of the store compared to the healthy product. Figure 3 (unhealthy product A) further demonstrates a steady baseline for the unhealthy product A, which is similar to what we found for the healthy product. For the unhealthy product B (Figure 4), on the other hand, the follow-up conditions are lower and steadier compared with the baseline conditions in period 4 and 5. This might be because the follow-up conditions are a “cleaner” “baseline,” as they were performed after a few weeks had lapsed from the experiment. As such the follow-up (baseline) is not under the threat of carry-over effects, as it seems like periods 4 and 5 (baseline) are sequentially confounded by the first successful interventions (placing the target product at the perimeter). This leads to an underestimation of the differences between the experimental intervention against the baseline.

We conclude that the results from the experiments clearly demonstrate that a floor display in the perimeter of a hypermarket store is very effective for all target products, both the healthy (Figure 2) and the unhealthy products (Figures 3, 4), but even more for the healthy product. As evident from Table 1, while an additional placement in the perimeter of the store increased the sales of the healthy product by 571% on average compared to baseline, the sale increase was 412 and 76% for the unhealthy products A and B, respectively. The daily total average sales in NOK were also highest for the healthy product in the alternating treatment condition but not in the baseline condition. Thus, we conclude that the effectiveness of the floor display in the perimeter of the store was higher for the healthy target product. If the retailer were mainly concerned about total revenues, it would be better to give additional floor space, from time to time, and in a high-traffic zone of the store, to such a healthy product, compared to very unhealthy chocolate bars.

**Table 1:** The results of a floor display in the perimeter compared to baseline (sales in NOK and percentage increase).

** Target product**	**Baseline**	**Intervention**	**Increase**	**Increase in**
	**(NOK)**	**(NOK)**	**in NOK**	**%**
Unhealthy product A	667	3,416	2,749	412
Unhealthy product B	1,528	2,684	1,156	76
Healthier product	547	3,672	3,125	571

The observational data in Table 2 provide some additional insight. The unhealthy product B seems to enjoy a much higher attentional impact on the consumers than the healthy target product. Nevertheless, the gap is almost closed regarding stop/hold power (the share of shoppers stopping/evaluating), and the healthy product outcompetes the unhealthy product in their respective closing powers (the share of shoppers purchasing from the display).

**Table 2:** Observational data for the healthy product and the unhealthy product B.

** Behavior**	**Healthier** **product**	**Unhealthy** **product B**
Noticing	49%	74%
Stopped/evaluating	23%	26%
Purchased	8%	6%
Number of observations	554	300

## 5. Conclusion and future directions for in-store impression management for healthy food marketing

### 5.1. Discussion and limitations

We have shown how impression management and environmental cues are used in stores to influence consumers' responses toward unhealthy and healthy food. We have given several examples and discussed how this marketing could be “turned around” from unhealthy food to healthier alternatives. It is important to systematically alter the circumstances in which consumer choices are made (point of purchase) by doing controlled experiments. Our showcase, involving a few in-store experiments with a brand that heavily uses history and tradition as part of its market communication, has given a clear example of how this research can be conducted. It showed clearly that a healthy food product can outperform a popular unhealthy product in terms of total sales increases, if placed in the perimeter of the store where most shoppers walk. Reach is the first essential step in the shopping process (37), and a product reaches most shoppers in the store when displayed along the perimeter of the store (30, 31, 37, 45). Thus, enhanced reach should, per se, lead to more sales of both the healthy product (fish balls) and the unhealthy products (chocolate bars), but the potential is higher for products with relatively more unfavorable locations in the store as a baseline condition (their regular placement in the store). As such, a more unfavorable baseline location, such as limited shelf space in a low-traffic area for the healthy product compared to a much higher shelf space for the unhealthy products in the convenience area of the store, gives the healthy product a relatively higher increase in reach than the unhealthy products, and thus a greater potential for increased sales. This is similar to what Sigurdsson et al. (32, 81) found in their in-store experiments involving replacing pastilles and chewing gum at the cash register with bananas and small packages of dried fish and fruit mixes. Their results showed that dried fish and fruit mixes enjoyed greater increases in sales than bananas since they had a more untapped potential due to their more unfavorable baseline locations.

Our showcase also indicates that products can have different stop/hold power and closing power when placed in a freestanding floor display at a high-reach location, which may help explain some of the results in our showcased experiments. Stop/hold power is the second crucial conversion element for in-store impression management, and in our showcase it reflects the impact of a floor display along the perimeter of the store on consumers' engagement with the display (37). Closing power, on the other hand, is the third and final conversion as it reflects the impact of the display on actual purchases (the share of zone visitors actually buying the displayed product). We found that the healthy product had a lower stop/hold power, but at the same time a higher closing power, than the unhealthy product B. This indicates that the healthy product is a niche product in the retail store; more of those seeing and engaging with it, buy it. More importantly, measuring stop/hold and closing power as part of in-store impression management gives a more fine-grained insight into the effectiveness of the in-store interventions.

This kind of functional analysis within stores should be developed further, as retailers can alter the surroundings to get consumers' attention, impress them, and create the desired consumer actions. This can be done from an evolutionary perspective (82) and ecology, with a focus on a natural science of behavior approach; changing one or more components of the functional unit of analysis—as has been depicted in the current paper. That is through responsible active retailing, direct manipulations (trying to grab attention and impress consumers), measures of the target behavior (in our case, buying behavior), as well as micro-conversions (or sub-goals: reaching and stopping consumers), leading to healthier food purchases. However, as illustrated in the experiments we have showcased, behavioral data are insufficient to provide all the insights needed for in-store impression management, as it mostly informs which interventions worked and which did not. In our showcase, the behavioral data indicate that the advertisement pointing to the brand's long tradition, and the background music designed to evoke associations with traditional food and thoughts of previous experiences with the brand, had no meaningful effect on the sales of the healthy target product. There can be many reasons for this. One plausible explanation is that the brand's iconic green tin can with fish balls makes consumers easily recognize the brand in the retail environment, which can activate thoughts about the healthy product (47). Thus, it is possible that a massive floor display of the product (without an ad or background music) has the ability to evoke positive associations, memories, and thoughts of previous experiences with the brand, and that an additional advertisement or background music does not add additional influence or impression to noticing the stack of the iconic tin cans themselves. It can also be that customers think, when noticing the massive floor display of fish ball cans, that this product was now discounted, especially since they are used to price promotions along the perimeter of the store (37), and have come to equate large quantities of a product displayed together with low price (83). The attractiveness of the floor display can also be a result of healthy food choices being more convenient (84), or vicarious learning; seeing other consumers shopping the target product, or as a social proof, seeing that it is being shopped in the presence of other shoppers (85). The point is that behavioral data have clear limitations as they do not address the underlying reasons for the behaviors.

By doing experiments, one finding can therefore lead to 10 more experiments/studies. It is, for instance, possible to theorize and test if an in-store environmental cue has impacted the image of a product or a brand to be viewed as more attractive, or differentiated (48), or if it activated certain thoughts about the brand. However, this would require a more profound approach also involving other types of data than behavioral data. As such, the in-store marketing level of analysis reveals the limits of the experimental analysis of behavior contribution to consumer psychology (see Foxall (86), Sigurdsson (87)], as our experimental extensional research [the objective concentration on linking behavior with environmental stimuli (88)] intentionally did not account directly for these speculations. It would have required going beyond observation and transactions as assessing cognitive and emotional reactions [e.g., perceptions and nostalgia—relying on intentional language and interpretation (88)] requires data gathered directly from shoppers who encounter the in-store stimuli. Testing the extent to which the responses are attributed to nostalgia, brand reputation, price/image, perceptions etc., would have to rely on method triangulation (33, 89); that is, the use of different types of methodology, data, and philosophy. In addition, such data can be used to better understand shoppers' behavioral responses (noticing, stopping/evaluating, purchasing) to the interventions.

### 5.2. Future directions

We emphasize studying behavior directly as it happens, especially as many behaviors can be somewhat unconscious, and it is well-documented that most consumers are not very equipped to predict their own behavior when it comes to self-control issues (81, 90, 91), such as eating healthily or exercising. The link between ideas and behaviors can be weak, and when it exists, it can be reciprocal. Consumers often come to know their beliefs and attitudes from what they do and choose (92). Our focus and showcase experiments focused on the importance of these direct measurements, as they tend to be underrepresented in the literature, at the expense of using questionnaires that are faster and simpler, not relying on cooperation with retailers. We clearly affirm that studying behavior in its natural habitat is essential. Despite this, we are convinced that impression management for healthy food marketing can also rely on indirect (e.g., questionnaires or interviews) measurements of behavior for deeper understanding and as a stimulation for further research. Doing interviews and questionnaires might be best at the point of purchase, that is, putting consumers in situations as realistic as possible. We showed that it is important to conduct a more fine-grained analysis of behavior by measuring not only the sales effects (in percentage and in economic value) in isolation, but also by going deeper into the behavioral conversions as a successful approximation of the final target behavior of buying; namely the healthy and unhealthy products' stopping and holding power (converting into a shopper) and their closing power (converting into a purchaser). These are the three levels of in-store marketing analysis as conversions for impression management. That is: (1) exploiting consumers' dominant paths (reach), (2) placements that impress consumers (stop/hold impressions), and (3) product attribute signaling (closing the sale).

### 5.3. Managerial implications

Our case experiments, and our studies in general, quoted in the current paper, demonstrate that retailers need to experiment with different products in the scarce and valuable area around the perimeter. Their experimental effort should include various products and focus on placements—making the product visible for attention and impression (as the best products tend to be at the best placements). The experiments we have showcased successfully identified one functional relation and, therefore, applied it to the problem of turning around the obesogenic retail environment where impression management and environmental cues can be used to influence consumers' responses toward healthier food selection. Along with making healthier food more convenient to purchase (better locations), there is also a need to conduct experiments testing the effects of various efforts intended to make healthier food more attractive to select. For instance, as Wansink (84) pointed out, fruits and vegetables could be more attractively named (e.g., using more descriptive names such as crisp carrots) and more attractive in appearance (e.g., fruit stacked on a flat table is less attractive than fruit on a more decorated display).

The promotional space along the perimeter of the store is rather scarce (93), meaning retailers possess a great extent of market power (29). Attractive placements in the stores are in great demand by brand suppliers, leading to a financial gain for the retailers through promotional allowances (93). Unless producers of unhealthy products pay substantially for the attractive floor space along the store's perimeter, grocery retailers can obtain financial gains from devoting more of this space to healthier food products at the expense of more unhealthy products, as we have showcased. However, since research indicates that store displays are predominately used for promoting unhealthy products (7), it is plausible to assume that brand suppliers of unhealthy products are able and willing to pay more in promotional allowances than suppliers of healthier products. This is a problem for the promotion of healthier food along the perimeter of the store. The UK has taken a clear stand on this issue by restricting the placement of unhealthy food and drinks at end-caps, or free-form displays close to end-caps pointing toward the perimeter. Such restrictions reduce the competition for promotional space and force retailers to use more of the displays in their customers' most chosen pathway to promote healthier food. This demands more insights that emphasize key environmental touch points throughout the customer journey in grocery retailing [in line with Larsen et al. (38)], analyzing the proportions of healthy vs. unhealthy store space (94) and monetizing every inch and possible cue within the store (37).

To conclude, in-store impression management research for healthy food should focus on the when, where, and how it is possible to present the retailer and the brands in the best light possible. Although it is important to market toward all five senses, the focus should be on visual elements: in sight (including peripheral vision) means in mind (95). Active retailers can use environmental cues at the beginning of the shopping journey. They can give a good first impression with a well-organized and theatrical fruits and vegetable section, strengthening the retailer's healthy and fresh image. The presentation and selection of carrying equipment at the entrance of the store (96) can also be applied here. Retailers can offer divided shopping carts at the entrance (97), implying social norms with a relatively large section of a shopping cart for fruits and vegetables. This intervention can affect the consumer throughout the whole shopping journey, even with smart shopping carts with digital screens promoting healthy choices. As our case experiments show, it is crucial to test how to apply the most important placements within the store (such as along the perimeter, using end-caps, and with large visual displays building on the principles of tonnage marketing in open spaces) and at the end of the journey (at the checkout). The marketing needs to be well-informed about consumer behavior, wants, and goals. Consumers often follow the lead of similar others or defer to experts who provide shortcuts to decision-making (85). It is therefore important to guide consumers in-store by using quality cues stemming from the action of other consumers, such as publishing product ratings (as peer opinions) and high sales/bestsellers (as peer behavior)—as Amazon has started to do in their Amazon 4-star physical stores. Stores should also establish more authority signals, recommending and guiding long-term happiness and health.

## Data availability statement

The datasets presented in this article are not readily available because it is data from a retailer and can therefore not be shared. Requests to access the datasets should be directed to nils.magne.larsen@uit.no.

## Ethics statement

Ethical review and approval was not required for the study on human participants in accordance with the local legislation and institutional requirements. Written informed consent for participation was not required for this study in accordance with the national legislation and the institutional requirements.

## Author contributions

NL, VS, and DG contributed to the conception and design of the study. DG collected the data. VS performed the statistical analysis. NL and VS wrote the first draft of the manuscript, while DG wrote sections of the manuscript. All authors contributed to the manuscript revision, read, and approved the submitted version.
